# Using singleton densities to detect recent selection in *Bos taurus*

**DOI:** 10.1002/evl3.263

**Published:** 2021-11-22

**Authors:** Matthew Hartfield, Nina Aagaard Poulsen, Bernt Guldbrandtsen, Thomas Bataillon

**Affiliations:** ^1^ Bioinformatics Research Centre Aarhus University Aarhus DK‐8000 Denmark; ^2^ Institute of Evolutionary Biology University of Edinburgh Edinburgh EH9 3FL United Kingdom; ^3^ Department of Food Science Aarhus University Aarhus N DK‐8200 Denmark; ^4^ Center for Quantitative Genetics and Genomics, Department of Molecular Biology and Genetics Aarhus University Tjele DK‐8830 Denmark; ^5^ Rheinische Friedrich‐Wilhelms‐Universität Bonn Institut für Tierwissenschaften Bonn DE‐53115 Germany; ^6^ Department of Veterinary Sciences Copenhagen University Frederiksberg C DK‐1870 Denmark

**Keywords:** *Bos taurus*, genomics, milk fat, milk protein, selection, stature

## Abstract

Many quantitative traits are subject to polygenic selection, where several genomic regions undergo small, simultaneous changes in allele frequency that collectively alter a phenotype. The widespread availability of genome data, along with novel statistical techniques, has made it easier to detect these changes. We apply one such method, the “Singleton Density Score” (SDS), to the Holstein breed of *Bos taurus* to detect recent selection (arising up to around 740 years ago). We identify several genes as candidates for targets of recent selection, including some relating to cell regulation, catabolic processes, neural‐cell adhesion and immunity. We do not find strong evidence that three traits that are important to humans—milk protein content, milk fat content, and stature—have been subject to directional selection. Simulations demonstrate that because *B. taurus* recently experienced a population bottleneck, singletons are depleted so the power of SDS methods is reduced. These results inform on which genes underlie recent genetic change in *B. taurus*, while providing information on how polygenic selection can be best investigated in future studies.

**Impact Statement**

Many traits of ecological or economic importance (including height, disease propensity, climatic adaptation) are “polygenic.” That is, they are affected by a large number of genetic variants, with each one only making a small contribution to a trait, but collectively influence variation. As selection acts on all of these variants simultaneously, it only changes the frequency of each one by a small amount, making it hard to detect such selection from genome data. This situation has changed in recent years, with the proliferation of whole‐genome data from many individuals, along with the development of methods to detect the subtle effects of polygenic selection. Here, we use data from 102 genomes from domesticated cattle (*Bos taurus*) that has experienced intense artificial selection since domestication, and test whether we can detect signatures of recent selection (arising up to 740 years ago). Domesticated species are appealing for this kind of study, as they are subject to extensive genome sequencing studies, and genetic variants can be related to traits under selection. We carried out our analysis in two parts. We first performed a genome‐wide scan to find individual genetic regions that show signatures of recent selection. We identify those relating to cell regulation, catabolic processes, neural‐cell adhesion, and immunity. In the second part, we then analyzed genetic regions associated with three key traits: milk protein content, milk fat content, and stature. We tested whether these regions collectively showed a signature of selection, but did not find a significant result in any of these cases. Simulations suggest that the domestication history of cattle affected the power of these methods. We end with a discussion on how to best detect polygenic selection in future studies.

Determining which genomic regions have been subject to selection is a major research goal in evolutionary genetics. Traditional methods have focused on detecting strong selection affecting individual genes (Nielsen, 2005; Vitti et al., 2013; Stephan, 2019). An alternative process is “polygenic selection,” where many loci contribute to genetic variation in a trait, so selection acting on it is expected to generate small and simultaneous allele frequency changes at multiple loci (Pritchard & Di Rienzo, 2010; Pritchard et al., 2010). Many polygenic models have been formulated to account for both the response to phenotypic selection and the maintenance of genetic variance in quantitative traits (reviewed by Sella and Barton [2019] and Barghi et al. [2020]). Among them is Fisher's infinitesimal model, which is important for its historical role in uniting population and quantitative genetics, and its recent renaissance in the context of genome‐wide association studies (Fisher, 1918; Barton & Keightley, 2002; Barton et al., 2017; Charlesworth & Edwards, 2018; Visscher & Goddard, 2019). Although it has been possible to identify which genetic regions contribute to trait variation, it has historically been hard to infer which alleles have been involved in the polygenic selection response. Extensive theoretical studies of how alleles at multiple loci act when a population adapts to a new optimum generally find that “large‐effect” alleles, which strongly affect a trait, are the first to spread and fix, whereas “small‐effect” alleles take much longer to reach high frequencies (de Vladar and Barton, 2014; Wollstein & Stephan, 2014; Jain & Stephan, 2015, 2017a, 2017b; Stetter et al., 2018; Thornton, 2019; Hayward & Sella, 2019). Furthermore, if epistasis exists between variants, many selected alleles do not reach fixation as they eventually become deleterious (de Vladar & Barton, 2014; Jain & Stephan, 2017b). The spread of large‐effect alleles may also be impeded if a faster adaptive response can be otherwise realized through changes at many small‐effect alleles (Lande, 1983; Chevin & Hospital, 2008; Pavlidis et al., 2012; Chevin, 2019). Alternatively, if the optimum shift is sufficiently big, then large‐effect mutations that first go to fixation can subsequently be replaced by small‐effect variants over longer timescales (on the order of the population size; Hayward and Sella [2019]). Overall, only a small proportion of loci affected by polygenic selection are expected to fix sufficiently quickly to leave selection signatures in genomic data (Pavlidis et al., 2012; Thornton, 2019).

Due to this difficulty, earlier methods for detecting polygenic selection focused on cases where selection favors distinct phenotypes in different populations, so trait differentiation among populations will be greater than expected under neutral drift. Tests for this form of selection relied on comparing *Q*_ST_ and *F*_ST_ statistics, which, respectively, measured mean genetic differentiation at the trait itself and a set of neutral loci (Whitlock, 2008; Le Corre & Kremer, 2012; Savolainen et al., 2013). Yet these methods do not determine which genomic regions are subject to selection. This situation has now changed with the increased number of genome‐wide association study (GWAS) data that link genotypes and phenotypes, as exemplified by the development of large cohort studies (e.g., the UK Biobank; Bycroft et al. 2018). The release of these data spurred a series of studies and new methods designed specifically to detect polygenic selection. These methods usually involve determining which SNPs affecting a phenotype show correlated changes in frequency (Berg & Coop, 2014; Racimo et al., 2018; Sanjak et al., 2018; Josephs et al., 2019; Berg et al., 2019a, 2019b; Uricchio et al., 2019; Edge & Coop, 2019; Kreiner et al., 2020; Wieters et al., 2021; Gramlich et al., 2021); which sets of alleles are associated with certain environmental or climatic variations (Coop et al., 2010; Turchin et al., 2012; Robinson et al., 2015; Yeaman et al., 2016; Exposito‐Alonso et al., 2018; Zan & Carlborg, 2018; Exposito‐Alonso et al., 2019; MacLachlan et al., 2021; Ehrlich et al., 2021; Fuhrmann et al., 2021; Rowan et al., 2021); or determining which SNPs or genetic regions explain a large fraction of phenotypic variance and trait heritability (Zhou et al., 2013; Yang et al., 2015; Gazal et al., 2017; Zeng et al., 2018; Schoech et al., 2019; Exposito‐Alonso et al., 2020; Duntsch et al., 2020; Zeng et al., 2021). Some of these approaches use overlapping methods.

Detecting recent polygenic selection is much harder, as long periods of time (number of generations on the order of the population size; Hayward & Sella, 2019; Thornton, 2019) may be needed to cause detectable frequency changes in alleles with small effect sizes. Over shorter timescales, these frequency changes are expected to be more modest and harder to detect (Stephan, 2016; Jain & Stephan, 2017a). A recent breakthrough in detecting these subtle changes was the development of the “Singleton Density Score” (SDS), a statistic tailored to detect recent and coordinated allele frequency changes over many SNPs (Field et al., 2016). Recent selection at a locus favoring one variant will lead to a reduction in the number of singletons (i.e., variants that are only observed once) around it. The SDS detects regions that exhibit a reduction in the density of singletons, to determine candidate regions that have been subject to recent selection. Using this approach, Field et al. (2016) found correlations between SDS scores at SNPs and their associated GWAS effect sizes for several polygenic traits in the modern UK human population, including increased height, infant head circumference, and fasting insulin. Their findings suggested that these traits have been subject to recent selection during the most recent 75 or so generations (about 2000 years). However, these (and other) results that detect selection for increased height may instead reflect previously unaccounted‐for population structure (Novembre & Barton, 2018; Barton et al., 2019; Sohail et al., 2019; Berg et al. 2019a; Uricchio et al., 2019; Edge & Coop, 2019).

The SDS method is ideally suited to organisms where large amount of whole‐genome data are available, along with quantitative trait loci (QTL) or GWAS information that link genotypes to phenotypes. Domesticated species are attractive systems for studying recent selection, as selected phenotypes are often already known and they are subject to large‐scale sequencing studies. Investigating the genetic architecture underlying rapid selection in these species is also important to determine how they respond to agricultural practices, and uncover selection targets that can be used to improve breeding programs (Georges et al., 2019). Domestic cattle *Bos taurus* has been subject to intensive genomics analyses to improve artificial selection for traits that are important for human use, including milk protein yield, milk fat content, and stature (Hayes et al., 2009; Meuwissen et al., 2013; Wray et al., 2019). These traits are influenced in part by an individual's genome, with significant heritability estimates being recorded, some as high as 80% (Soyeurt et al., 2007; Haile‐Mariam et al., 2013; Buitenhuis et al., 2016). Previous selection scans on *B. taurus* reported individual regions that were likely to be subject to recent selection, some of which were close to genetic regions for stature, milk protein content, and milk fat content (Lemay et al., 2009; MacEachern et al., 2009; Qanbari et al., 2010; Boitard & Rocha, 2013; Qanbari et al., 2014; Zhao et al., 2015; Boitard et al., 2016a; Bouwman et al., 2018). However, these traits are polygenic, with several genetic regions and QTLs associated with each (Lemay et al., 2009; Boitard et al., 2016a; Bouwman et al., 2018; van den Berg et al., 2020). Although recent methods have been developed to detect polygenic environmental adaptation (Rowan et al., 2021), there has yet to be a formal test of whether these intrinsic traits show evidence of polygenic selection.

Here, we applied the SDS method to whole‐autosome sequencing data from 102 *B. taurus* Holstein individuals. We first determined genetic regions that have been subject to recent directional selection, and subsequently tested whether evidence exists for recent selection acting on a set of QTLs underlying either milk protein content, milk fat content, or stature in this breed.

## Results

### METHODS OUTLINE

We filtered the data to retain only biallelic SNPs that had a sensible level of coverage and did not lie in putatively over‐assembled regions (i.e., duplicated sections that caused many reads to assemble at a specific genetic location). Over‐assembled regions appear as highly heterozygous with elevated coverage, and can exhibit false signatures of recent selection. We also obtained a set of singletons and filtered them to retain high‐quality variants where both alleles were equally well covered to remove potentially erroneous calls. We polarized test SNPs using outgroup sequences and applied the SDS test of Field et al. (2016) to detect recent selection, with increased SDS values reflecting selection favoring derived SNPs over ancestral variants. We standardized SDS scores with those of a similar frequency, so they are normally distributed (similar normalization was also carried out by Field et al. 2016). These values are denoted sSDS for “standardized SDS.” Further details are available in the *Methods* in the Supporting Information.

### ESTIMATING TIMESCALE OF SELECTION

We first determined the timescale over which we expect to detect selection in *B. taurus* using the SDS method. SDS measures the changes in singleton numbers around putatively selected SNPs, relative to background numbers in the absence of selection. As singletons arise on the tips of the underlying gene trees, the average tip length in the genealogy of sequenced samples determines the timescale over which the SDS detects a signal (Field et al., 2016). As more haploid genomes are included in the study, the time to first coalescence between two samples decreases, reducing the tip lengths and therefore shortening the timescale over which SDS detects selection (Field et al., 2016). We hence simulate tip‐ages over a range of sample sizes to investigate how this timescale changes accordingly.

To calculate the mean tip age, we simulated gene genealogies under two scenarios. We first simulated the Holstein population demography inferred by Boitard et al. (2016b), which suggested that this population experienced a sudden decline in effective population size (*N*_e_) since domestication, but with a present‐day *N*_e_ (∼793) that is much larger than that inferred from pedigree data (∼49; Sørensen et al. 2005) or from temporal variation in SNP frequencies (∼48; Jiménez–Mena et al., 2016). Hence, we also simulated genealogies under a second model that used the Boitard et al. (2016b) demographic model, but with the present‐day *N*_e_ set to 49. These scenarios will be referred to as the “High *N*_0_” and “Low *N*_0_” models, respectively.

**Figure 1:**
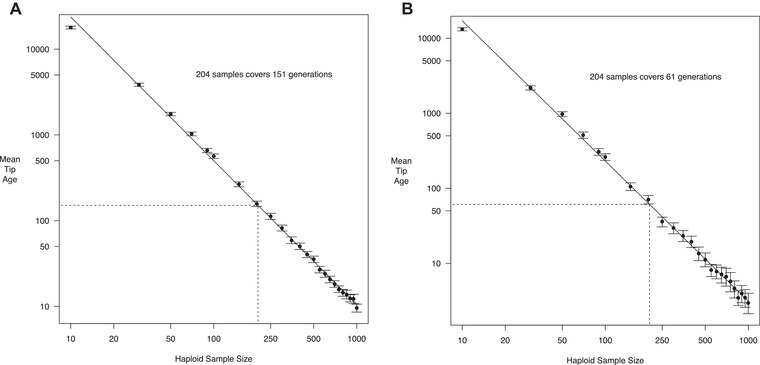
Simulated mean tip age for *B. taurus*, as a function of the number of haploid samples. Simulations assumed either (A) demography as inferred by Boitard et al. (2016b) (the “High *N*_0_” model) or (B) the same but with a smaller present‐day *N*_e_ of 49 (the “Low *N*_0_” model). Points are the mean values; bars show 95% confidence intervals. The solid line is the best linear fit to the log of both values; dotted lines show the predicted tip age for 204 alleles.

Figure 1 shows simulation results. Depending on the assumed present‐day *N*_e_, the tip length in our sample of 204 alleles (i.e., assuming two per diploid individual) goes back either 65 or 148 generations. Assuming 5 years per generation (Boitard et al., 2016b), this timescale corresponds to between 325 and 740 years ago. Since *B. taurus* domestication started around 10,000 years ago (Zeder, 2008), the sample size used in this study will only capture selection acting in the very recent past that is more relevant for breed formation, rather than selection during *B. taurus* domestication. Sample sizes and tip‐ages are linearly related on a log‐log scale, meaning that an increase in sample size will greatly decrease the timescale over which SDS detects selection. For example, with 500 haplotypes then SDS will detect selection acting no more than 50 generations ago, depending on the underlying demographic model.

We will focus on detecting selection signatures assuming the high *N*_0_ model. Results using the low *N*_0_ model to calibrate scores were broadly similar. They are outlined in the Supporting Information; we will highlight when differences arise.

### GENOME‐WIDE sSDS

**Figure 2:**
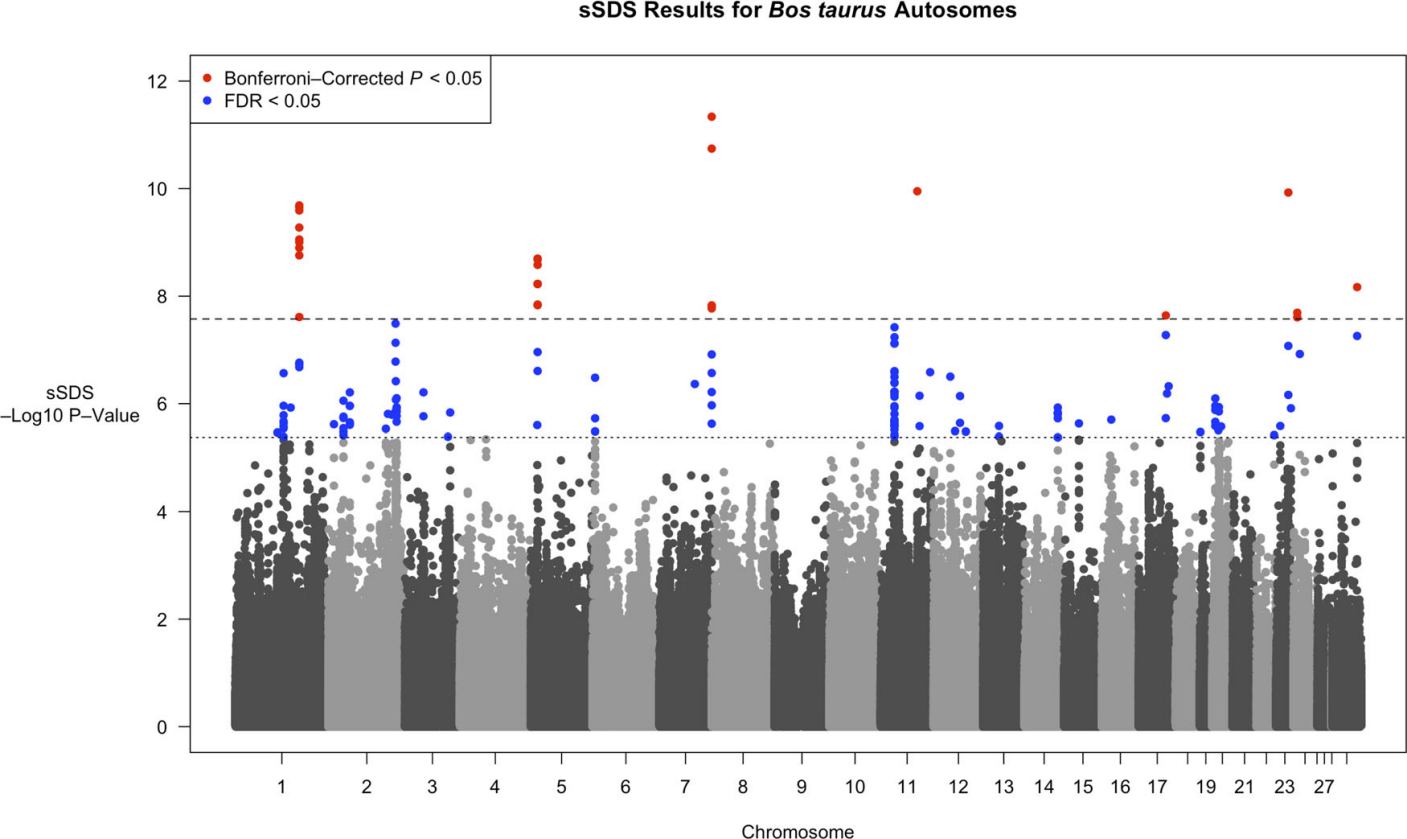
*P*‐values of sSDS scores across *B. taurus* autosomes, as plotted on a negative Log_10_ scale, as a function of the chromosome. Alternating black and gray points show (nonsignificant) values from different chromosomes. Blue points are SNPs with FDR < 0.05, with the cutoff denoted by a horizontal dotted line. Red points are SNPs with Bonferroni‐corrected *P*‐value < 0.05 (actual *P*‐value < ∼2.7 × 10^−8^), with the cutoff denoted by a horizontal dashed line. Figure S1 shows results for the Low *N*_0_ model.

Figure 2 plots sSDS values (at SNPs with minor allele frequency greater than 5%) across all autosomes, excluding chromosome 25 (due to an insufficient number of singletons needed to obtain SDS scores after filtering). Many SNPs have elevated sSDS scores (158 SNPs at FDR < 0.05; 306 for the low *N*_0_ model). Several regions contain SNPs with significantly high sSDS values (Bonferroni‐corrected nominal *P* < 0.05; actual *P* < ∼2.7 × 10 ^−8^). To further investigate potential selection targets, we looked for genes that either overlapped significant SNPs or lay 10‐kb up‐ or downstream of them. Linkage disequilibrium (LD), as measured by *r* ^2^, decays to around 0.2 over 50 kb in Danish Holstein breeds (Buitenhuis et al., 2016), so genes within 10 kb should be in LD with regions harboring high sSDS scores. Table 1 lists these genes, with more targets present under the low *N*_0_ model. Most of these genes are of unknown function (as listed on UniProt); the list also includes an snRNA. *PPM1L* is involved with cellular regulation and the activation of stress‐activated protein kinases. *TDO2* is involved in tryptophan‐related catabolic processes, whereas *NTM* is implicated in neural cell adhesion. SNPs with significantly elevated scores are also found on chromosome 23 near the MHC region, which may reflect overdominant selection. All Bonferroni‐significant SNPs were removed from subsequent tests of recent polygenic selection to prevent directional selection from skewing the underlying sSDS distributions. Figure S1 shows results for the low *N*_0_ model.

**Table 1:** Genes that overlap or lie close to Bonferroni‐significant sSDS regions. The “High, Low *N*_0_” column specifies which genes are close to significant SNPs for each *N*_0_ model

Chromosome	Gene Name	Start Position	End Position	Gene Biotype	High, Low N _0_
1	PPM1L	106405113	106727070	Protein Coding	High, Low
5	TMCC3	24306913	24595494	Protein Coding	High, Low
5	CEP83	24070404	24345243	Protein Coding	High, Low
17	U6	43381106	43381209	snRNA	Low
17	CTSO	43364999	43381605	Protein Coding	Low
17	TDO2	43386894	43403747	Protein Coding	High, Low
23	OR12D2H	29291787	29292713	Protein Coding	High, Low
23	OR12D2E	29305933	29309785	Protein Coding	High, Low
24	GAREM1	24694637	24927333	Protein Coding	High, Low
29	NTM	34576918	34994005	Protein Coding	High, Low

### TESTING FOR POLYGENIC SELECTION ACTING ON MILK FAT, PROTEIN AND STATURE

If polygenic selection were acting on specific traits, we expect a positive correlation between the effect sizes of variants underpinning a trait, and selection acting on them as measured by sSDS. We collated sSDS scores of SNPs that lie close to QTLs reported for either milk fat percentage, milk protein percentage (van den Berg et al., 2020), or those that lie close to stature QTLs (Bouwman et al., 2018). The latter were inferred from a meta‐analysis of GWAS studies conducted in seven Holstein populations, but not every QTL had an effect size reported in each population. We hence investigated two overlapping consensus QTL sets, where an effect size was either reported in at least six of seven populations (yielding 42 QTLs with sSDS scores associated with them) or where effect sizes were reported in at least five of seven populations (58 QTLs had sSDS scores). We repolarized SDS scores so that a positive score reflected a trait‐increasing effect; we denote these values “tSDS” following Field et al. (2016). We then determine if there was a positive correlation between the absolute log_10_‐value of the QTL *P*‐value (a proxy for the effect size) and tSDS.

**Figure 3:**
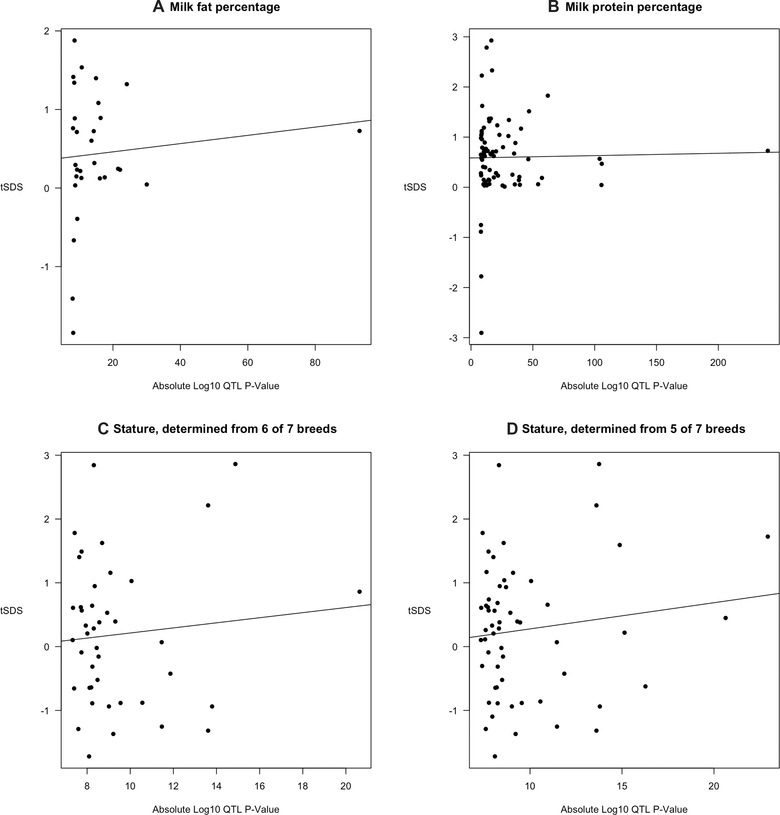
Relationship between tSDS scores near milk or stature QTLs, as noted in the subheadings, and the absolute log *P*‐value of QTLs. Lines show a linear model regression fit. Figure S3 shows results assuming a low *N*_0_ model.

Figure 3 shows the relationship between QTL *P*‐values and tSDS for SNPs that lie close to QTLs. Although positive trends are observed as determined using a linear model, they all exhibit nonsignificant correlations (milk fat percentage Spearman *ρ* = 0.0990, *P* = 0.603; milk protein percentage Spearman *ρ* = 0.0354, *P* = 0.758; stature from six breeds Spearman *ρ* = −0.0739, *P* = 0.642; stature from five breeds Spearman *ρ* = −0.00966, *P* = 0.943). Relationships remain nonsignificant after removing an outlier point for the milk traits whose QTL has an extremely low *P*‐value (Figure S2), and also under the low *N*_0_ model (Figure S3; see figure legends for correlation *P*‐values).

sSDS (and tSDS) can become correlated along the genome if focal SNPs are in LD with one another, which was not accounted for in the preceding analyses. To determine whether LD could have affected these correlations, we randomly subsampled sSDS scores from SNPs that shared the same chromosome and bin of derived‐allele frequency as the SNPs used in the above analyses, and repolarized them to transform them into tSDS values. We then determined the Spearman's *ρ* associated with these permuted values to determine whether that for the true data was significantly elevated (see *Methods* for details). In all cases, the observed value was not significantly higher than for permuted values (see Figure S4 for histograms and exact *P*‐values, which all exceed 0.05). We therefore conclude that these QTL datasets do not harbor SNPs with significantly different tSDS scores compared to the rest of the genome.

## Discussion

### SUMMARY OF RESULTS

We analyzed an extensive *B. taurus* genomic dataset to identify signatures of recent selection in the Holstein breed, and to determine whether the data contained a signal of polygenic selection acting on milk proteins and QTLs underlying phenotypic variation in stature. Given the sample size and the demographic history of Holsteins, the SDS method can detect very recent selection events arising no more than approximately 740 years ago (Figure 1). A whole‐genome scan for sSDS scores identified several targets of recent directional selection that overlap or lie close to protein‐coding genes (Figure 2; Table 1). The genes whose functions are known are involved in protein regulation, catabolic processes, and neural‐cell adhesion. Significant values were also observed near the MHC region. We subsequently investigated whether either milk protein genes or SNPs near stature QTLs collectively showed evidence of polygenic selection. We did so by testing whether there is a relationship between the QTL effect size, as measured by its *P*‐value, and tSDS values to SNPs near them. However, no relationship was observed, even after performing a permutation test (Figs. 3 and S2‐S4). Hence, although sSDS could reveal specific instances of recent selection, tests based on collective scores of variants associated with known selected traits yielded no signal of polygenic selection.

### POTENTIAL REASONS FOR A LACK OF POLYGENIC SELECTION SIGNAL

#### Impact of Holstein demographic history

Although the SDS method detected individual candidate genes for very recent selection, we were unable to find strong evidence for polygenic selection acting on three traits that were subject to artificial selection since domestication. This result is *a priori* surprising, given that these traits have been subject to recent intense artificial selection. Recent studies generally find nonzero heritability estimates for them, indicating that there should be the potential for genetic variants underpinning them to change in response to artificial selection (Soyeurt et al., 2007; Haile‐Mariam et al., 2013; Buitenhuis et al., 2016). In addition, the ratio of the mutation and recombination rates in cattle is just over three (Boitard et al., 2016b; Harland et al., 2018), indicating that several informative SNPs exist per haplotype that should improve the power of the SDS method (in contrast, this ratio is approximately equal to one in humans; Field et al., 2016).

One potential reason for this lack of signal is due to the population history of *Bos taurus*. The effective population size of many *B. taurus* breeds appears to have undergone a decline since domestication (Sørensen et al., 2005; Boitard et al., 2016b), which likely reflects successive bottlenecks due to domestication, breed formation, and intense recent selection. Population size reductions are known to reduce the number of low‐frequency variants and increase the prevalence of intermediate‐frequency variants (Harpending et al., 1998), which can affect the power of the SDS method. To understand if the history of *B. taurus* affects the detection of recent selection in Holstein cattle using SDS, we ran coalescent simulations to determine its ability to detect ongoing selection, given realistic Holstein population history and genetic parameters (see *Methods* for details). We simulated a partial sweep occurring in the middle of a 10‐Mb region, either assuming a mutation rate in line with what has been inferred for Holstein or one 10‐fold higher to replicate diversity expected in a genetic region with an elevated mutation rate.

**Figure 4:**
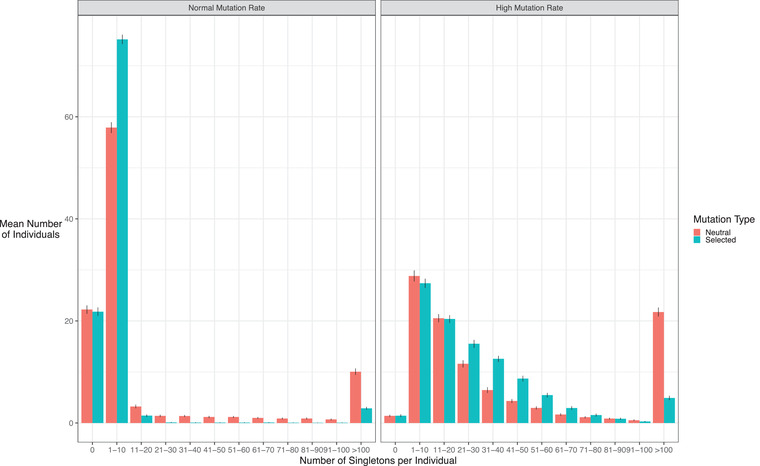
Mean distribution of singleton numbers per individual for each simulation, either assuming a standard mutation rate (left) or a 10‐fold higher mutation rate (right). Bars represent 95% confidence intervals.

For the standard mutation rate, no SDS scores were produced for any simulations. After inspecting the simulation results, we see that there is a large skew in the distribution of singleton numbers per individual with a large number of individuals (over 20 on average) that do not carry singletons at the end of simulations, preventing the calculation of a local SDS score (Figure 4). This fraction remained the same irrespective of whether the simulated region was neutral or subject to selection; the main effect of a sweep was to reduce the mean number of singletons per individual, which is the signal measured by SDS (Field et al., 2016). This reduction in overall singleton numbers is consistent with the known effects of population size contraction on reducing tip lengths (Harpending et al., 1998).

**Figure 5:**
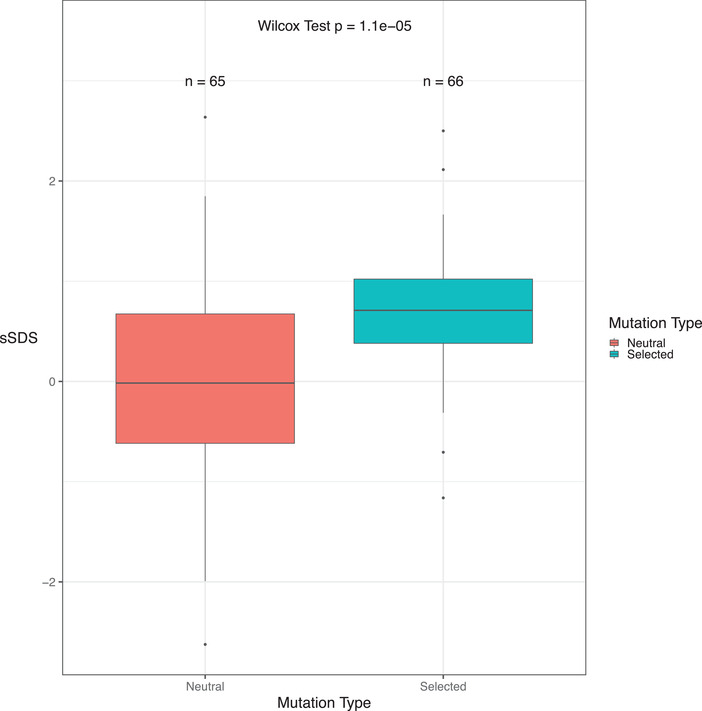
Distribution of simulated sSDS scores assuming a high mutation rate, for the neutral and selected cases. Numbers above each box plot denote how many simulations produced SDS scores and were included in the plot.

With a 10‐fold higher mutation rate, there were fewer cases where no individual harbored singletons (Figure 4). Accordingly, SDS scores could be calculated for 65 and 66 out of 100 simulations for the neutral and selective cases, respectively. In these cases, sSDS values were significantly higher in the selected case than for the neutral case (Figure 5; two‐sided Wilcox Test *P* = 1.1 × 10^−5^). However, note that sSDS values is less than one for the selected case, which does not exceed the FDR threshold in our study (for the high *N*_0_ case, the smallest sSDS value with FDR < 0.05 is 4.46).

Although singleton numbers differ between the two cases, a reduction in power could also be caused by a more general reduction in diversity due to the small recent effective population sizes of cattle. To investigate this effect, we estimated the fixed *N*_e_ that would yield the same number of segregating sites in simulations using the standard mutation rate, based on Watterson's estimator (Watterson, 1975; Hudson, 1990; see *Methods* for details). In both cases where selection is present or absent, *N*_e_ estimates lie at around 25,000, which is that inferred at approximately halfway between the onset of domestication and the present day (Boitard et al., 2016b; Figure S5). Given that estimates are similar irrespective of whether a sweep was present or not, the reduced population size caused by domestication could have also affected power due to limiting genetic variation and thus the potential to detect subtle sweep signatures associated with polygenic selection.

Overall, these simulations are consistent with population size reductions in *B. taurus* both reducing the overall genetic diversity and the number of singletons, which limits its ability to detect partial sweeps. SDS is more likely to detect signals in regions of elevated mutation rate, suggesting there will likely be an ascertainment bias in where signals are detected in the genome. The reduction in singletons also reduces the power to investigate SDS values in telomeric regions. SDS values are calculated using the distance up‐ and downstream from a SNP to the nearest singleton, and are undefined if a certain number of samples do not harbor singletons in either direction (Field et al., 2016). SDS values are hence less likely to be defined in telomeric regions, as it is generally less feasible to observe singletons up until the end of the chromosome. This problem is exacerbated if there are few singletons overall.

#### Other potential reasons for a lack of signal

Another potential reason for a lack of signal is that the selection response on these traits may have been driven by large‐effect variants that have already fixed in the population, with a smaller contribution from small‐effect mutations. Theoretical models have shown that more major‐effect QTLs are likely to fix if the population lies further from a fitness optimum (Lande, 1983; Jain and Stephan, 2017b; Thornton, 2019). Domesticated species, which experience strong and sustained directional artificial selection, especially in recent generations, could thereby fix more adaptive mutation via sweep‐like processes compared to populations evolving in more stable environments (Lande, 1983; Jain & Stephan, 2017a). Furthermore, once a population has adapted to a new environment (the domestication phenotype in this case), then any remaining major‐effect mutations are likely to be superseded by variants with weaker effects, which are harder to detect (Hayward & Sella, 2019). The response to polygenic selection will be further weakened in smaller populations (John and Stephan, 2020), which could be a factor given the reduced effective population sizes of *B. taurus* (Sørensen et al., 2005; Boitard et al., 2016b). There is some evidence of this explanation; selective sweeps signatures are associated with stature QTLs (Bouwman et al., 2018), and the study of van den Berg et al. (2020) was more likely to identify milk QTLs that had a moderate to high minor allele frequency, suggesting reduced power to detect low‐frequency variants that are potential contributors to polygenic selection. Conversely, the stature meta‐analysis by Bouwman et al. (2018) found significant SNPs that explained up to 13.8% of the variance in stature, which is similar to that explained by significant SNPs for human height (16%), which is a classic trait for polygenic selection studies. Hence, there may be sufficient polygenic SNPs present to test for polygenic selection, but the power will still be reduced due to the demographic history of Holstein cattle.

Potential solutions to increase power include increasing sample sizes; using alternative methods; or analyzing different kinds of genome data to detect polygenic selection. Applying SDS to a larger sample size would increase the power to detect selection acting in the recent past (Figure 1; see also Field et al. 2016), but overall power will still be limited by the tip‐length of neutral genealogies. Recent developments in methodology involve directly inferring trees from genome data, and using these to identify subtle sweep signatures associated with trait variants (Edge & Coop 2019; Speidel et al., 2019; Stern et al., 2021). These methods have greater power to detect weakly selected mutations that may be segregating for longer than the tip‐length of the population.

Another approach would be to look beyond sequence data and focus on gene networks (reviewed by Fagny and Austerlitz 2021). The recently proposed “omnigenic” model (Boyle et al., 2017; Liu et al., 2019) posits that variation in quantitative traits is principally affected by a plethora of “peripheral” genes that indirectly affect them, rather than a limited set of “core” genes that directly modify a trait. These numerous peripheral genes may exert their influence via regulatory effects (e.g., gene expression changes), but are also expected to be highly pleiotropic. Fully testing the omnigenic model will require larger datasets and novel experimental designs (Wray et al., 2018). A recent example is from an experiment with *Drosophila melanogaster*, where gene knockouts that do not pass a GWAS significance threshold for pupal length still significantly affect it (Zhang et al., 2021). There is also nascent evidence that gene regulation may underlie directional polygenic selection. Boitard et al. (2016a) found that some adaptive signatures of *B. taurus* are located in intergenic regions; regulatory changes were also proposed to guide polygenic selection in *Arabidopsis* (He et al., 2016). Analyses of gene sets associated with infection responses or immunity also found evidence for polygenic selection in humans and primates (Daub et al., 2013, 2017; Svardal et al., 2017). Immunity gene sets might be exceptional cases, as they are more likely to contain genes subject to very strong selection (Castellano et al., 2019). Further investigations using regulatory information and a broader range of gene sets could be a promising approach to determine the impact of polygenic selection.

## Materials and Methods

Full methods are available in the Supporting Information.

## AUTHOR CONTRIBUTIONS

All authors contributed to the study design. NAP and BG provided data. MH performed the analyses and wrote the manuscript, with feedback from NAP, BG, and TB.

## DATA ARCHIVING

Raw SDS scores and polarization information have been deposited on Dryad (https://doi.org/10.5061/dryad.547d7wm8q). Data analysis and simulation scripts are available on GitHub (https://github.com/MattHartfield/CattleSDS).

## CONFLICT OF INTEREST

The authors declare no conflict of interest.

Associate Editor: Z. Gompert

## Supporting information

Figure S1: Genome wide sSDS results for the low *N*_0_ model.
Figure S2: Polygenic selection test results for the high *N*_0_ model (milk fat percentage, milk protein percentage) after removing outlier point.
Figure S3: Polygenic selection test results for the low *N*_0_ model.
Figure S4: Permutation test results.
Figure S5: *N*_e_ estimates from simulations.
Figure S6: Histogram of mean *F*‐values for each individual.
Figure S7: Schematic of data filtering.
Figure S8: Density of mean site depth.
Figure S9: Example of sliding‐window analysis to detect over‐assembled regions (OARs).
Table S1: List of putative OARs.
Table S2: Cutoff values for filtering singletons.
